# Upregulator of Cell Proliferation Predicts Poor Prognosis in Hepatocellular Carcinoma and Contributes to Hepatocarcinogenesis by Downregulating FOXO3a

**DOI:** 10.1371/journal.pone.0040607

**Published:** 2012-07-16

**Authors:** Chan Xie, Li-bing Song, Jue-heng Wu, Jun Li, Jing-ping Yun, Jia-ming Lai, Dong-ying Xie, Bing-liang Lin, Yun-fei Yuan, Mengfeng Li, Zhi-liang Gao

**Affiliations:** 1 Department of Infectious Diseases, the Third Affiliated Hospital of Sun Yat-Sen University, Guangzhou, Guangdong Province, China; 2 Department of Experimental Research, Sun Yat-sen University, Guangzhou, China; 3 Department of Microbiology, Zhongshan School of Medicine, Guangzhou, Guangdong, China; 4 Department of Biochemistry, Zhongshan School of Medicine, Guangzhou, Guangdong, China; 5 Department of Pathology, Cancer Center, Sun Yat-sen University, Guangzhou, China; 6 Department of hepatobiliary surgery, the First Affiliated Hospital of Sun Yat-sen University, Guangzhou, Guangdong Province, China; 7 Department of Hepatobiliary Surgery, Sun Yat-sen University Cancer Center, Guangzhou, China; 8 Key Laboratory of Tropical Disease Control, Ministry of Education, Sun Yat-sen University, Guangzhou, Guangdong Province, China; University of Hong Kong, Hong Kong

## Abstract

**Objective:**

The goal of the present study was to investigate the potential correlation between the expression level of upregulator of cell proliferation (URGCP/URG4) and the prognosis of hepatocellular carcinoma (HCC), and to examine the biological function of URGCP/URG4 in the progression of HCC, to better understand its underlying molecular mechanism in hepatic tumorigenesis.

**Design:**

URGCP/URG4 expression was analyzed in 15 HCC cell lines, in 278 archived paraffin-embedded HCC sections, and in 10 pairs of fresh HCC tumor and para-tumor non-cancerous tissues using immunohistochemistry (IHC) and Western blotting analysis (WB). The effect of URGCP/URG4 on cell proliferation and tumorigenesis was examined in vitro and in vivo. WB and luciferase reporter analyses were performed to identify the effects of URGCP/URG4-overexpression or -knockdown on expression of cell cycle regulators and transcriptional activity of FOXO3a.

**Results:**

IHC results revealed an upregulation of URGCP/URG4 in all HCC cell lines and fresh HCC samples as compared with normal liver cells and para-tumor tissues, respectively. URGCP/URG4 was also expressed at a high level in 122 of the 278 (43.8%) archived HCC specimens. The expression level of URGCP/URG4 was significantly correlated with clinical staging and poor patient survival of HCC in the study cohort, and in various clinical subgroups. Strikingly, ectopic expression of URGCP/URG4 induced proliferation and anchorage-independent growth of HCC cells, while silencing of URGCP/URG4 had the opposite effect. Furthermore, URGCP/URG4 overexpression in HCC cells increased cellular entry into the G1/S transitional phase, associated with downregulation of p27^Kip1^ and p21^Cip1^ and upregulation of cyclin D1. These effects were accompanied by enhanced Akt activity and reduced FOXO3a transcriptional activity.

**Conclusions:**

URGCP/URG4 plays an important role in promoting proliferation and tumorigenesis of HCC and may represent a novel prognostic biomarker and therapeutic target for this disease.

## Introduction

Hepatocellular carcinoma (HCC) is the fifth most commonly diagnosed cancer and the third major cause of cancer-related death in the world [1]. HCC represents a particularly serious health problem in Asia, where in association with high rates of hepatitis B virus (HBV) infection, its incidence in Eastern and Southeast Asian countries is as high as 35.4 and 18.3, and 12.6 and 5.7 per 100,000 male and female inhabitants, respectively [2]. It has been recognized that for more than 75% of Asian HCC cases, chronic HBV infection contributes to its etiology, and high serum viral load is predictive of HCC development [3]. Despite the improvement made in treatment strategies for HCC during recent decades, the disease continues to have a high mortality rate, mainly due to late diagnosis and lack of effective therapies for advanced HCC [4]. Surgery has been established as one of the most effective therapeutic modalities for patients with good liver function; however, frequent post-resection recurrence of HCC remains a major clinical problem [5], with the 5-year survival rate after curative resection being only 35%–43% [6], [7]. It is of note, that patients with HCC of the same clinical stage have variable survival rates, thus improved indicators of disease development are of important clinical value for accurate evaluation of patient prognosis [8], [9].

It is currently challenging to identify HCC patients with a good prognosis after curative surgery, particularly in those with early-stage disease who do not demonstrate vascular invasion, regional lymph nodes, or distant metastases [10]. Clinically, the prognosis of an HCC patient closely depends on both the clinicopathological features of the tumors, and the liver function of the patient. While several staging systems are available for classifying HCC, they have limitations for determining clinical outcomes, especially in patients with early-stage disease [11]. Examples of commonly used scoring systems for evaluating HCC prognosis include the Cancer of the Liver Italian Program (CLIP) and the Japan Integrated Staging (JIS) scoring system [12], [13]. These multi-parameter scoring systems, however, are either insufficiently effective in predicting the prognosis of patients diagnosed as early-stage HCC, or impeded by high false-negative and false-positive rates associated with inclusion of α-fetoprotein (AFP) in the prognostic parameter system [14], [15]. Hence, identification of novel prognostic molecular markers could facilitate differentiating clinical outcomes in patients with a given stage of disease and improve the selection of patients for adjuvant therapies after surgical resection [16].

It is widely recognized that the X protein of HBV (HBx) is essential for viral replication and contributes to hepatocarcinogenesis [17]. In this context, interest has been attracted to identifying genes whose expression is responsive to HBx. One such gene is upregulator of cell proliferation (URGCP), which had previously been named HBx upregulated gene 4 (URG4), and was originally identified as a gene inducible in HCC cells by transfection with HBx [18]. Overexpression of URGCP/URG4 in HCC and gastric cancer cells was found to promote cell growth and survival in tissue culture and soft agar; however, the in vivo effect of URGCP/URG4 on HCC remains unknown [18], [19]. Furthermore, it has been shown that overexpression of URGCP/URG4 in HCC and gastric cancer cells upregulated cyclin D1, whereas repression of URGCP/URG4 downregulated it. Moreover, overexpressed URGCP/URG4 in osteosarcoma tissues has been linked to tumor recurrence and metastasis, as well as the proliferative activity of osteosarcoma cells, suggesting that URGCP/URG4 may be a valuable prognostic marker for certain types of human cancer [20]. Interestingly, amplification of cyclin D1 gene expression, and overexpression of cyclin D1 protein has been detected in HCC, suggesting a potentially important link between URGCP/URG4, cell cycle regulation and HCC progression; though the molecular mechanism of how URGCP/URG4 regulates the expression of cyclin D1 is unclear [21], [22]. As such, understanding the mechanisms underlying the proliferative actions of URGCP/URG4 might help develop novel anti-HCC strategies and new prognostic biomarkers for HCC. In the current study, we report correlated URGCP/URG4 expression with clinical staging and patient survival in HCC. Our mechanistic data also indicate that the Akt/FOXO3a signaling pathway might play a role in mediating the proliferative effect of URGCP/URG4 in HCC cells.

## Methods

### Normal Liver Samples

Normal liver samples were collected from patients undergoing resections of hepatic hemangiomas at the Department of Hepatobiliary Surgery, the First Affiliated Hospital of Sun Yat-sen University, upon approval by Sun Yat-sen University First Affiliated Hospital Internal Review Board. Samples were collected and analyzed with written informed consent.

### HCC Specimens

A total of 278 paraffin-embedded, archived HCC specimens, which were histopathologically and clinically diagnosed as HCC at the Sun Yat-sen University Cancer Center from 1995 to 2003, were used in the current study. The clinicopathologic characteristics of the 278 patients is summarized in Table S1. Fresh HCC tissue samples, together with their paired adjacent non-cancerous tissues from each patient, were collected from HCC curative resection surgery, snap frozen and stored at −80°C until use for experimental purposes. For the use of above clinical materials, prior patients’ consent and approval from the Institutional Research Ethics Committee were obtained. HBV infection was diagnosed when HBV surface antigen (HBsAg) was detected by ELISA in the serum isolated from peripheral blood. All the patients enrolled in this study were HCV negative.

### Cell Lines

HCC cell lines, including QGY-7703, QGY7701, SMMC7721, HepG2, Hep3B, PLC/PRF/5, HuH7, HepG2215, HCCC9810, Bel-7402, Bel-7404, HCCL-M3, HCCL-M6, MHCC-97L and MHCC-97H, were obtained from the Sun Yat-sen University Cancer Center and grown in Dulbecco’s modified Eagle’s medium, supplemented with 10% fetal bovine serum, 2 mM L-glutamine, 100 units/ml penicillin and 100 ug/ml streptomycin (Invitrogen, Carlsbad, CA). Immortalized normal liver epithelial cells THLE3 were maintained in bronchial epithelial growth medium, supplemented with 5 ng/ml epithelial growth factor, 70 ng/ml phosphoethanolamine and 10% fetal bovine serum, at 37°C in a humidified atmosphere containing 5% CO_2_ (Clonetics Corporation, Walkersville, MD).

### IHC

IHC procedure to detect URGCP/URG4 and Ki67 and scoring of URGCP/URG4 expression were performed as previously reported [23]. IHC staining was quantitatively analyzed with the AxioVision Rel.4.6 computerized image analysis system assisted with automatic measurement program (Carl Zeiss, Oberkochen, Germany). Briefly, the stained sections were evaluated at 200× magnification, and ten representative staining fields of each section were analyzed to produce Mean Optical Density value (MOD), which represented the strength of staining signals as measured per positive pixels. The MOD data were statistically analyzed using *t*-test to compare the difference of average MOD between different groups of tissues.

### Luciferase Assay

Cells were transfected with p3× IRS-MLP-luc plasmid using a standard protocol. In brief, one hundred nanograms of p3× IRS-MLP-luciferase plasmid or control-luciferase plasmid plus 10 ng pRL-TK renilla plasmid (Promega, Madison, WI) were transfected into HCC cells using the Lipofectamine 2000 reagent (Invitrogen Co., Carlsbad, CA). Luciferase and renilla signals were measured 48 h after transfection using the Dual Luciferase Reporter Assay Kit (Promega, Madison, WI) according to a protocol provided by the manufacturer. Transfection was done in duplicates and repeated at least three times in independent experiments.

### Xenografted Tumor Model

Fourteen male NOD/SCID mice (4 weeks of age, 12–14 g) were purchased from the Institute of Materia Medica (Chinese Academy of Sciences, Beijing, China). This study was carried out in accordance with the recommendations in the Guide for the Care and Use of Laboratory Animals of the National Institutes of Health. The protocol was approved by the Institutional Animal Care and Use Committee of Sun Yat-Sen University. All surgery was performed under anesthesia with sodium pentobarbital. Mice were divided into two groups. For the QGY-7703/URGCP/URG4 group (n = 7), QGY-7703/URGCP/URG4 or QGY-7703/pMSCV-Vector cells, respectively, were subcutaneously inoculated on the right or left flank of each mouse. For the QGY-7703/URGCP/URG4 RNAi group (n = 7), QGY-7703/URGCP/URG4 RNAi or QGY-7703/pSuper-Vector cells, respectively, were inoculated on the right or left flank of each mouse. Before inoculation, all cells were suspended in PBS at a concentration of 1 × 10^7^/100 µl PBS. After inoculation, tumor growth was examined every three days by measuring the length and width with a caliper, and tumor volumes were calculated as Length×Width^2^×0.52. On day 36, animals were euthanized, and tumors were excised and weighed.

Other experimental procedures are available in Materials and Methods S1.

### Statistical Analysis

All statistical analyses were carried out using the SPSS v. 13.0 statistical software packages. Comparisons between groups for statistical significance were performed with two-tailed paired Student’s *t* test. The correlation between URGCP/URG4 expression and clinicpathological characteristics was analyzed uising chi-square test. Survival curves were plotted by the Kaplan-Meier method and compared using the log-rank test. Survival data were evaluated by using univariate and multivariate Cox regression analyses. A *P* value <0.05 was considered statistically significant in all cases.

## Results

### Upregulation of URGCP/URG4 in HCC Cell Lines and Tumor Tissue

**Figure 1:**
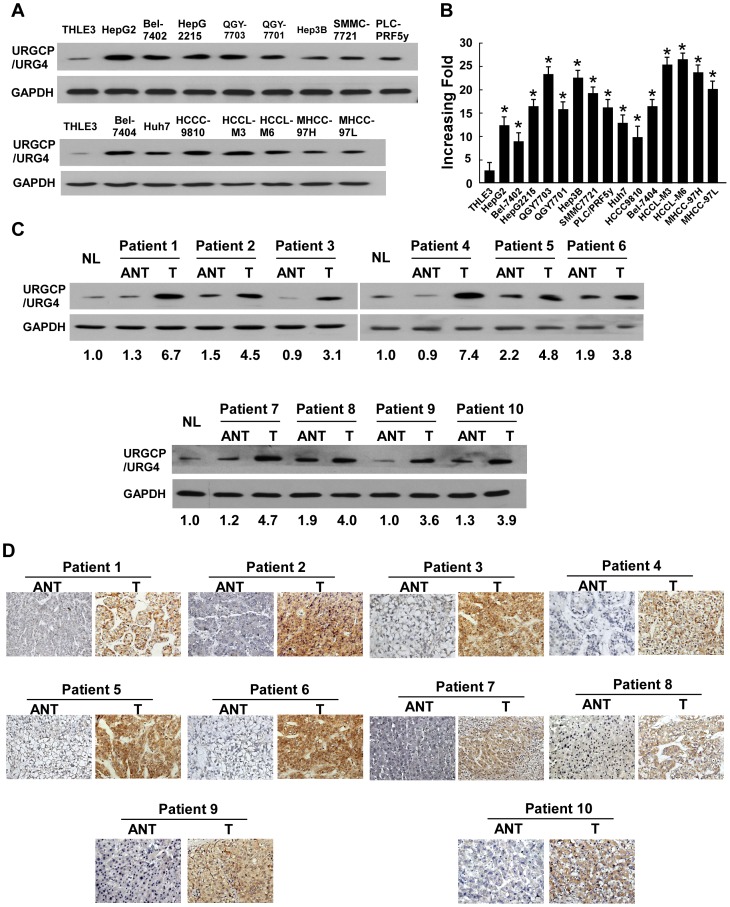
URGCP/URG4 is elevated in HCC. Expression of URGCP/URG4 protein (A) and mRNA (B) in normal human liver epithelial cells (THLE3) and cultured HCC cell lines QGY-7703, QGY-7701, SMMC-7721, HepG2, Hep3B, PLC/PRF/5, HuH7, HepG-2215, HCCC-9810, Bel-7402, Bel-7404, HCCLM3, HCCLM6, MHCC97L and MHCC97H. GAPDH was used as a loading control. Western blotting (C) and IHC (D) analysis of URGCP/URG4 protein expression in each of the tested primary HCC tissues (T) and adjacent non-cancerous tissues (ANT) taken from the same patient. Error bars represent SD from three independent experiments. *P<0.05.

To investigate whether URGCP/URG4 might play a role in the development and progression of HCC, we analyzed its expression at the protein and mRNA level. Our results revealed significantly higher levels of URGCP/URG4 expression in all 15 HCC cell lines examined, as compared to the level in the immortalized normal liver epithelial cell line, THLE3 (Figure 1A and 1B). To determine whether URGCP/URG4 upregulation found in HCC cell lines was clinically correlated with HCC progression, its expression was analyzed in HCC and paired non-cancerous tissues collected adjacent to cancerous lesions; with each pair taken from the same patient. URGCP/URG4 was found to be overexpressed at both the mRNA and protein level in all 10 human primary HCC samples examined, as compared with the expression level in adjacent tissue (Figure 1C). In agreement with our Western blot data, IHC analysis also showed high expression of URGCP/URG4 overexpression in all 10 tumors (Figure 1D).

### Association Between URGCP/URG4 Expression and Clinical Features of HCC

**Figure 2:**
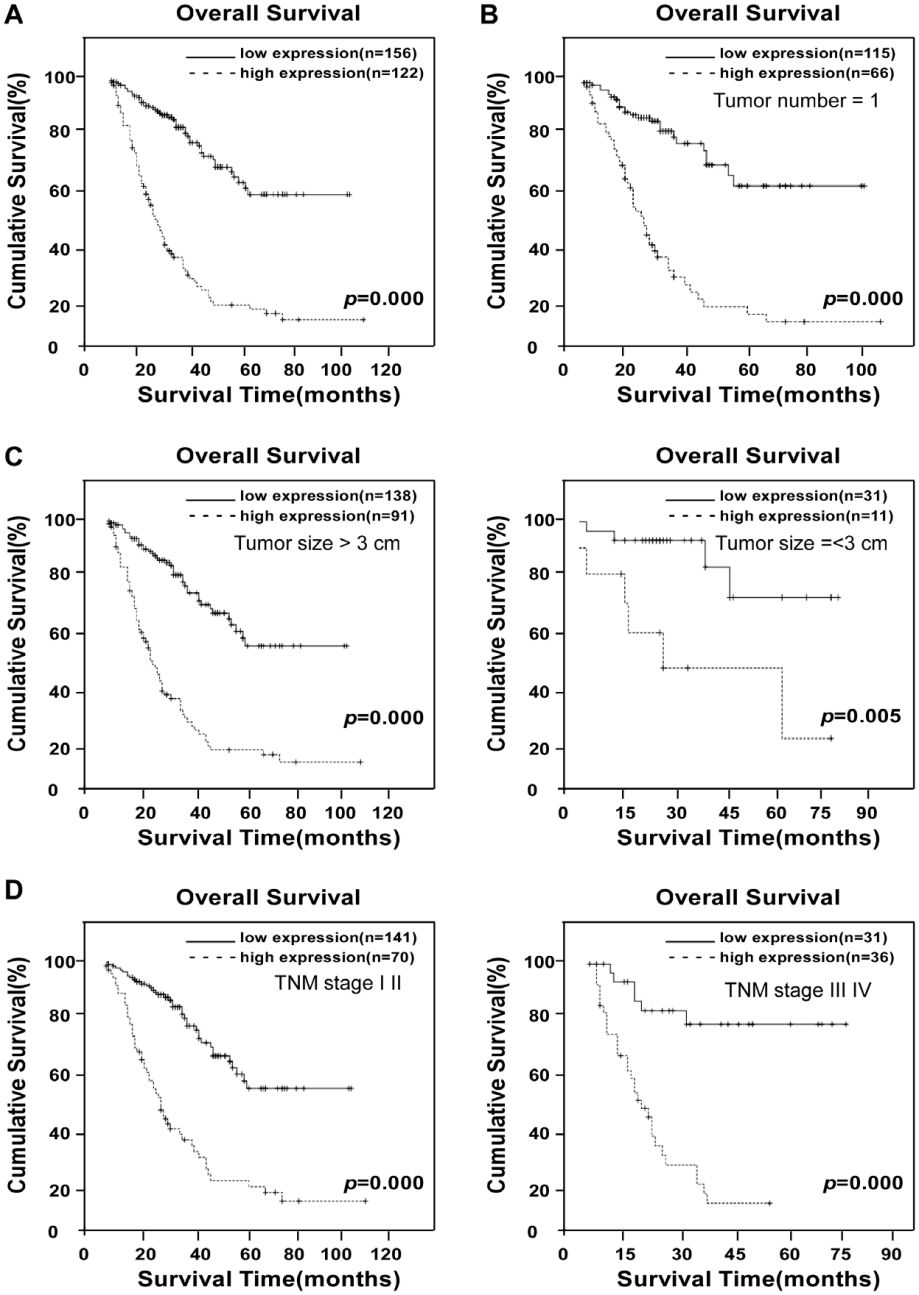
Kaplan-Meier analysis of OS in 278 cases based on URGCP/URG4 expressions in HCC clinical subgroup. Kaplan–Meier curves with univariate analyses (log-rank) for patients with low- versus high-URGCP/URG4 expression (A). OS in patients with a single tumor lesion (B). When patients were divided into subclinical groups according to tumor size, probabilities of survival with either HCC lesion diameter >3 cm (left) or ≤3 cm (right), high- and low-URGCP/URG4 expression was found to distinguish lower and higher SO rates, respectively (C). OS rates in patients subgrouped into TNM stage I-II (left) and TNM stage III-IV (right) as differentiated by high- or low-URGCP/URG4 expression (D).

To investigate the clinical relevance of the observed upregulation of URGCP/URG4 expression in HCC, the correlation between URGCP/URG4 expression level and the clinicopathological features of 278 HCC cases was retrospectively examined by IHC. This cohort included 16 cases of TNM stage I (5.8%), 195 cases of stage II (70.1%), 61 cases of stage III (21.9%) and six cases of stage IV HCC (2.2%; Table S1). Of the 278 HCC patients enrolled in this study, data of HBV tests were available for 261 cases, and HBV infection was detected in 229 (87.7%). As shown in our (Figure S1A and S1B), URGCP/URG4 protein expression was predominantly detected in the cytoplasm of tumor cells, and the expression of URGCP/URG4 was significantly higher in cancer lesions than in the adjacent non-cancerous tissues (*P*<0.001). URGCP/URG4 expression in tumor tissues was determined as strong (score >6) in 122 cases (43.9%) and weakly positive (score 0 to 6) in 156 cases (56.1%; Table S1). Quantitative analysis of IHC staining indicated that URGCP/URG4 expression in primary tumors (clinical stages I to IV) was statistically higher than that in the adjacent non-cancerous tissues (*P*<0.05, Figure S1A and S1B). Interestingly, our IHC analysis showed that URGCP/URG4 was drastically upregulated in HCC lesions excised from patients of late-stage HCC (TNM stages III to IV) as compared with those in the early stages of HCC, and that the URGCP/URG4 expression level correlated with TNM staging (Table S2). Furthermore, the survival time and vital status of HCC patients also correlated with URGCP/URG4 expression level (*P*<0.001 Figure 2A; Table S2). Kaplan-Meier and log-rank survival analyses on the cohort were performed to further demonstrate whether URGCP/URG4 expression was predictive of patient survival. As shown in Figure 2A, the survival time was found to be significantly different between low- and high-URGCP/URG4-expressing patient groups (*P*<0.001). We also noted that in this cohort, the AFP biomarker was not predictive of survival time (Figure S1C). While the 3-, 5- and 9-year overall survival (OS) rates for the whole study population were 45%, 36% and 33%, respectively, the OS rate in the low URGCP/URG4-expressing group increased to 68%, 57% and 57%, respectively. Whereas, the 3-, 5- and 9-year OS rates for the high URGCP/URG4- expressing group, were as low as 14%, 10% and 7%, respectively. Thus, the URGCP/URG4 expression level was found to be a prognostic factor for the overall survival of HCC patients (*P*<0.001, Table S3).

When the OS of patients in different clinical subgroups were further investigated in the same cohort, it was found that in the subgroup of patients with low AFP (< = 400 ng/mL), low URGCP/URG4 expression predicted a 5-year OS rate of 75.7%, as compared with 12.5% for the high-URGCP/URG4 group (*P*<0.001, Figure S1D). In the clinical subgroup with a single HCC lesion, the 5-year survival rates were 60.5% and 14.2% for low- or high-URGCP/URG4 expressing patients, respectively (*P*<0.001, Figure 2B). In the group of patients whose tumor sizes were ≤3 cm in diameter, the 5-year survival rate was 73.7% in the low-URGCP/URG4 group, as opposed to 49.1% for patients exhibiting high URGCP/URG4 expression (*P*<0.005, Figure 2C). With regard to URGCP/URG4 expression in early-stage HCC patients (TNM stages I–II), analysis of patients with low URGCP/URG4 expression revealed a 5-year survival rate of 52.4%, whereas the survival rate decreased to 17.6% in the high-URGCP/URG4 group (*P*<0.001, Figure 2D). Taken together, we interpret our data to suggest that URGCP/URG4 expression has potential prognostic value for HCC patients in various clinical subgroups (Table S4).

### Knockdown of URGCP/URG4 Impairs Cell Proliferation and Tumorigenicity in Vivo

**Figure 3:**
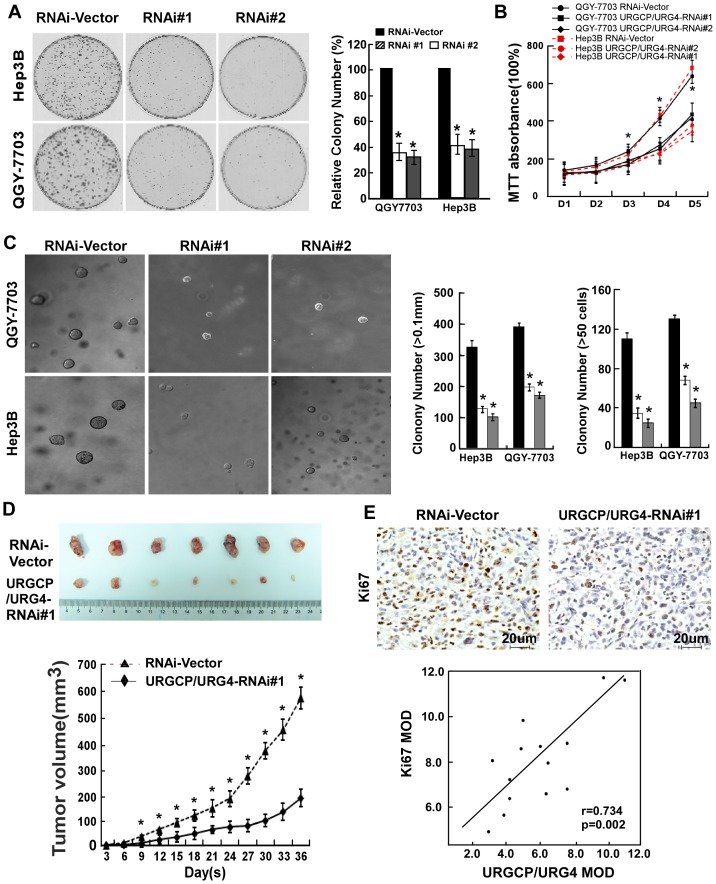
URGCP/URG4 is essential for HCC cell proliferation. Representative micrographs (left panel) and quantification (right panel) of crystal violet-stained cells (A). Silencing endogenous URGCP/URG4 inhibited cell growth, as determined by MTT assay (B). Silencing endogenous URGCP inhibited cell growth, as determined by anchorage-independent growth assay (C). Soft agar colony formation by indicated cells after 10 days of culture (left panel); colonies larger than 0.1 mm in diameter (middle panel) or those contained more than 50 cells/colony (right panel) were scored. Each bar represents the mean ± SD of three independent experiments. Xenograft model in NOD/SCID mice (D). QGY-7703/vector cells and QGY-7703/URGCP/URG4 RNAi#1 cells were injected into the groin of athymic mice and excised at experimental endpoint (upper panel). Tumor volumes were measured on the indicated days. Representative graph of tumor growth and mean tumor weights 5 weeks after inoculation are shown (lower panel). All data are shown as mean ± SD. IHC staining of URGCP/URG4 and Ki-67 in mouse HCC specimens (E). IHC analyses were performed for two independent times on each of the samples (upper panel); a significant correlation between URGCP/URG4 and Ki-67 levels was observed (*r* = 0.734; *P*<0.005; lower panel).

While previous studies have shown that overexpression of URGCP/URG4 promoted HepG2 cell growth, and that knockdown of URGCP/URG4 appeared to inhibit the proliferation of HCC cells in vitro, it is not known whether endogenous URGCP/URG4 contributes to the growth of HCC tumors in vivo. In the current study, we employed two HCC cell lines to express ectopic URGCP (Figure S2A), or to silence URGCP/URG4 expression, using two different specific shRNAs. We first confirmed that URGCP/URG4 regulated HCC cell proliferation and the cell cycle, as demonstrated by colony formation assay (see Materials and Methods S1, Figure S2B and Figure 3A), MTT assay (see Materials and Methods S1, Figure S2C and Figure 3B), soft agar (see Materials and Methods S1, Figure S2D and Figure 3C), BrdU staining (see materials and methods S1, Figure S2E and Figure S4A) and cell cycle analysis (see materials and methods S1, Figure S3A, Figure S4B and S4C). To further evaluate the in vivo effect of URGCP/URG4 on HCC growth, URGCP/URG4-overexpressing or -silenced QGY-7703 cells were employed in a xenograft mouse model and evaluated for tumor growth. Our results demonstrated significantly accelerated growth of tumors derived from URGCP/URG4-overexpressing QGY-7703 cells, as compared to the pMSCV vector-controlled cells, from day 12 after implantation through to the experimental endpoint at day 36 (*P*<0.05; Figure S3B and S3C). It is of particular note that for QGY-7703 HCC cells with URGCP/URG4 silenced, xenografts exhibited a significantly decreased rate of growth, as evidenced by lower tumor volume and weight (Figure 3D). Specifically, the mean tumor volume at the end of the experiment was 589.06±39.1 mm^3^ for control QGY-7703 cells, versus 188.43±43.0 mm^3^ for QGY-7703/URGCP-silenced cells, and the corresponding mean tumor weight was of 466.43±47.2 mg and 195.57±34.1 mg, respectively (*P*<0.05). IHC assessment of excised xenograft tissue sections showed that the fraction of proliferating cells were significantly reduced in QGY-7703/URGCP/URG4-silenced tumors in comparison with those in the transfection vector-controlled xenografts, with Ki67 MOD value of 7.66±1.45 and 9.19±1.02, respectively (Figure 3E and Figure S3D). Interestingly, we also observed that the expression of URGCP/URG4 was in close correlation with Ki67 staining intensity in HCC lesions (Figure 3E). Thus, our in vivo data suggests that URGCP/URG4 has a prominent proliferative and pro-tumorigenic effect in HCC.

### URGCP/URG4 Downregulates the Cell-cycle inhibitors p21^Cip1^ and p27^Kip1^ and Upregulates the Cell Cycle Regulator Cyclin D1

**Figure 4:**
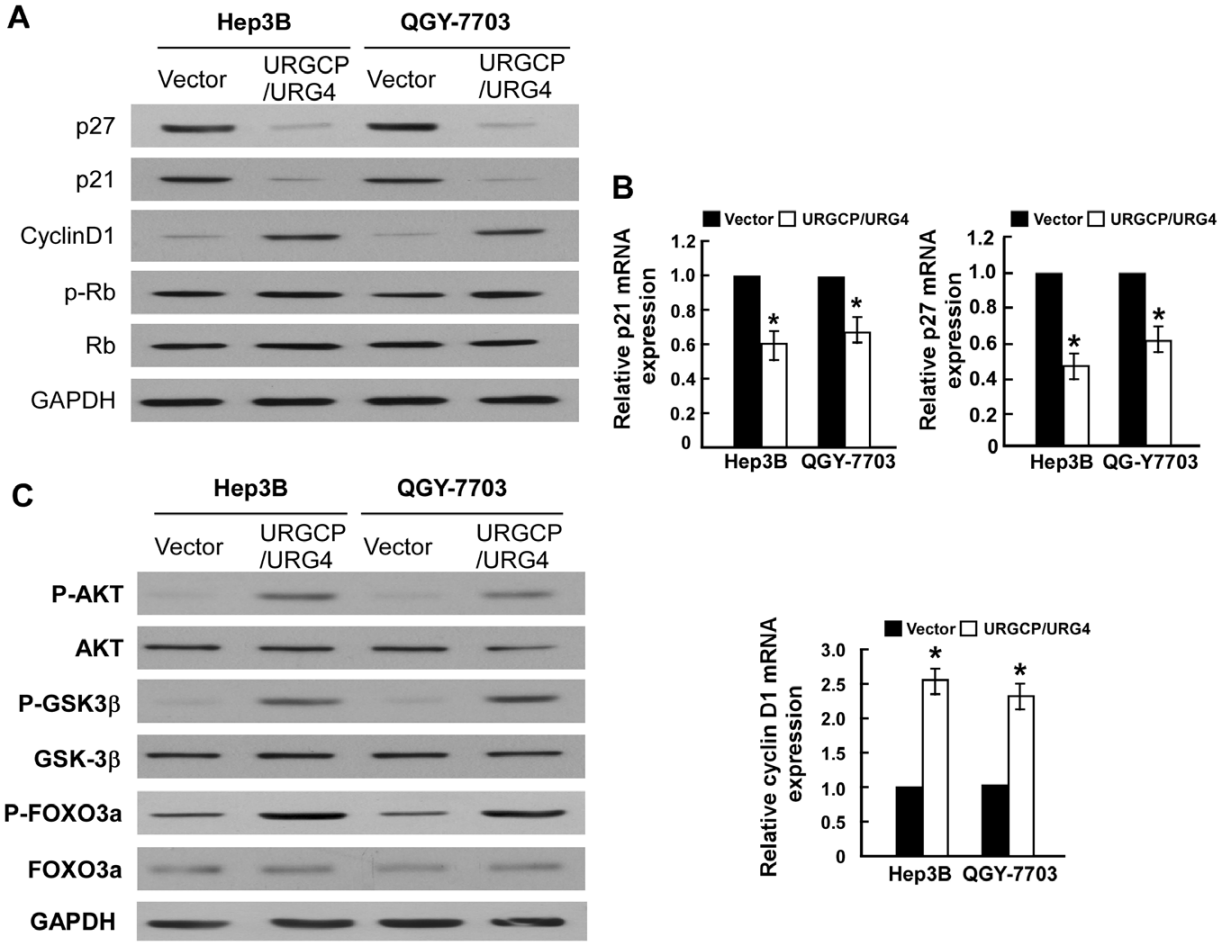
URGCP/URG4 induces proliferation through regulating CDK and CDK inhibitors. Western blotting analysis of expression of p21^Cip1^, p27^Kip1^, cyclin D1, phosphorylated Rb (p-Rb), and total Rb protein in indicated HCC cells (A). Real-time PCR analysis of expression of p21^Cip1^, p27^Kip1^, cyclin D1 in indicated cells (B). Western blotting analysis of phosphorylated Akt (p-Akt), total Akt, phosphorylated GSK-3β (p-GSK-3β), total GSK-3β, phosphorylated FOXO3a (p-FOXO3a-Ser253) and total FOXO3a proteins in indicated HCC cells (C). GAPDH was used as a loading control for all Western blots and mRNA expression levels were also normalized to GAPDH.

**Figure 5:**
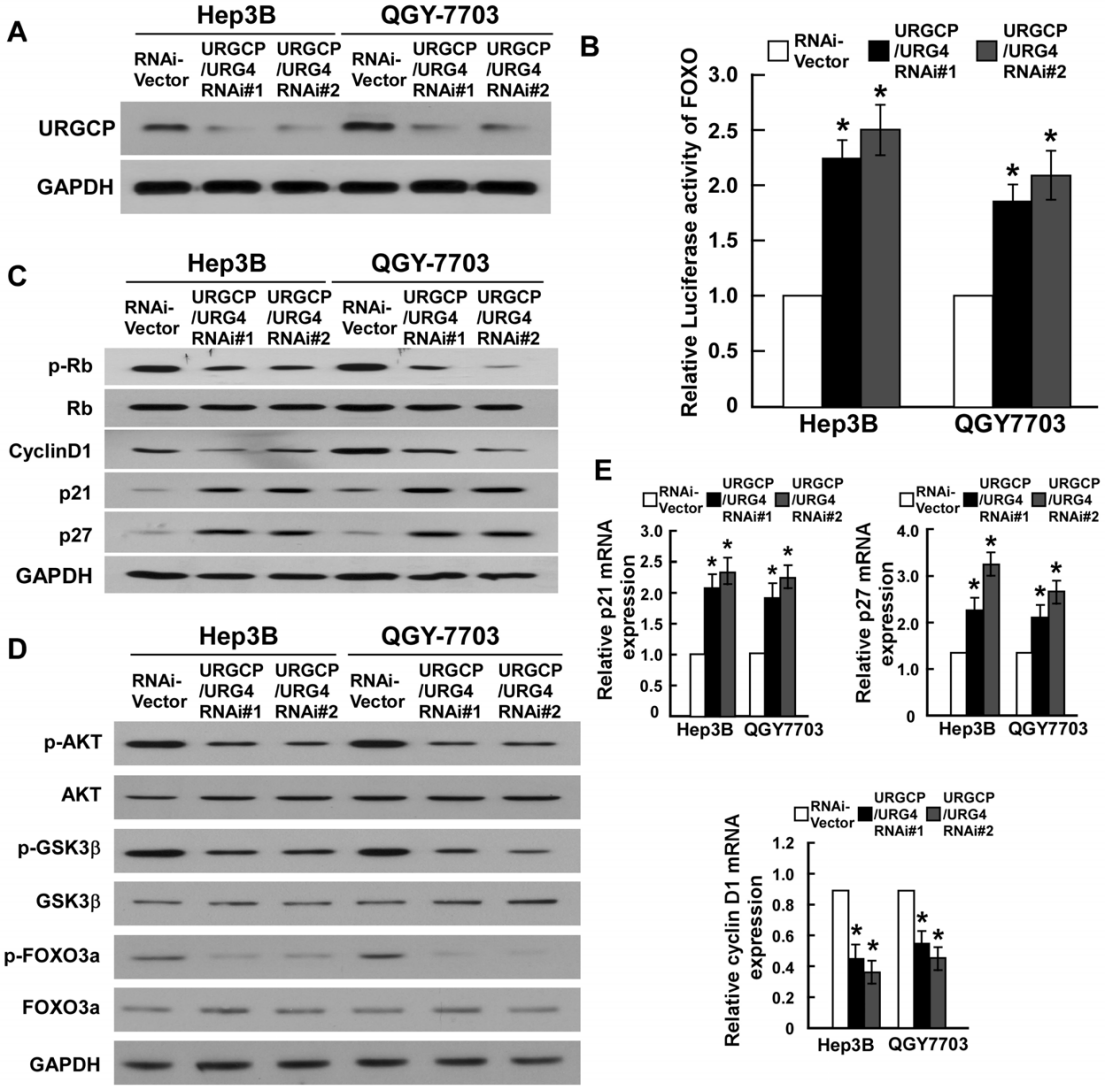
Downregulation of URGCP/URG4 enhances transcriptional activity of FOXO3a. Knockdown of URGCP/URG4 in two specific shRNA-transduced HCC cell lines (A). Relative FOXO3a reporter activity in cells transduced with vector, URGCP/URG4 shRNA#1 or URGCP/URG4 shRNA#2 (B). Western blotting analysis of phosphorylated Rb (p-Rb), total Rb, p21^Cip1^, p27^Kip1^ and cyclin D1 proteins in indicated HCC cells (C). Western blotting analysis of phosphorylated Akt (p-Akt), total Akt, phosphorylated GSK-3β (p-GSK-3β), total GSK-3β, phosphorylated FOXO3a (p-FOXO3a-Ser253) and total FOXO3a proteins in indicated HCC cells (D). Relative mRNA expressions of p21^Cip1^ (left panel), p27^Kip1^ (right panel) and cyclin D1 (lower panel) in indicated HCC cells were determined by real time RT-PCR (E). GAPDH was used as a loading control for all Western blots and mRNA expression levels were also normalized to GAPDH. Error bars represent SD from three independent experiments. *P<0.05.

We further examined the levels of cell cycle regulators in URGCP/URG4-transduced HCC cells at the protein and mRNA level, and found that p21^Cip1^ and p27^Kip1^ were downregulated, whereas the expression of cyclin D1 was upregulated in URGCP/URG4-overexpressing cells, in comparison with control cells (Figure 4A and 4B). We also observed increased phosphorylation of Rb, which is a downstream target of cyclin-dependent kinases (CDK), a result which corresponds with the changes in cell-cycle regulators seen (Figure 4A to 4C). The regulatory effect of URGCP on CDK was further investigated in URGCP/URG4-silenced cells (Figure 5A), in which we saw a dramatic upregulation of p21^Cip1^ and p27^Kip1^ protein and mRNA, while the expression of cyclin D1 was downregulated in comparison to control cells (Figure 5B to E). Taken together, these results suggest that the expression of cell cycle regulators are under the control of URGCP/URG4.

### FOXO3a is Involved in URGCP/URG4-induced Proliferation of HCC Cells

**Figure 6:**
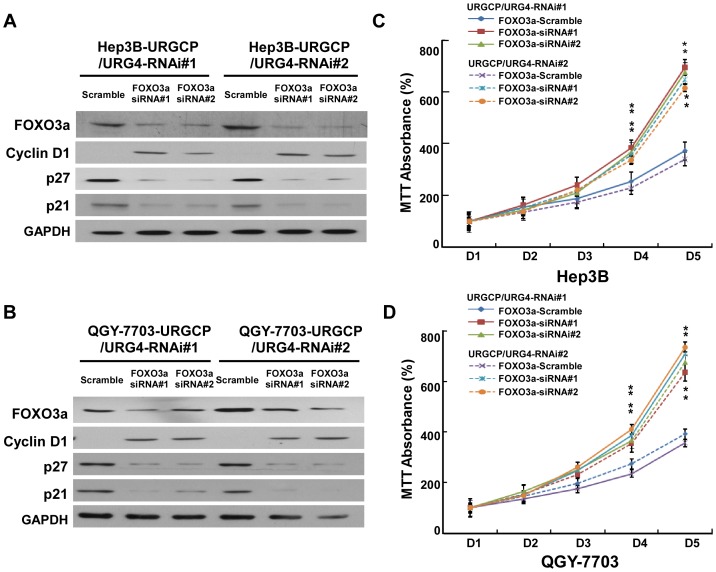
Silencing URGCP/URG4 expression impaired cell proliferation through FOXO3a. Western blotting analysis of p21^Cip1^, p27^Kip1^, cyclin D1 and FOXO3a proteins in indicated Hep3B (A) and QGY-7703 (B) cell lines. Silencing FOXO3a increased the proliferation of URGCP/URG4 shRNA(s)-transduced cells as determined by MTT assay in Hep3B (C) and QGY-7703 (D) cells. GAPDH was used as a loading control for all Western blots. Error bars represent SD from three independent experiments. *P<0.05.

It has previously been established that the transcription factor FOXO3a regulates the transcription of p27^Kip1^, p21^Cip1^ and cyclin D1 [24], as such we asked whether FOXO3a was involved in mediating the observed URGCP/URG4-induced increase in cell proliferation. Our results demonstrated that phosphorylation of FOXO3a and its upstream kinase, Akt, was abrogated when URGCP/URG4 expression was silenced, which occurred in parallel with an increase in p27^ Kip1^ and p21^Cip1^ and decreased cyclin D1 (Figure 5C to E). By contrast, overexpressing URGCP/URG4 in HCC cells elevated Akt and FOXO3a phosphorylation (Figure 4C). Furthermore, in the URGCP/URG4-silenced HCC cells, when FOXO3a was further knocked down, p27^ Kip1^ and p21^Cip1^ expression could be suppressed and the expression of cyclin D1 was restored (Figure 6A and B). Moreover, MTT assay showed that knocking down FOXO3a reversed the inhibitory effect of URGCP/URG4-knockdown on cell proliferation (Figure 6C and 6D), suggesting an important role of the Akt/FOXO3a axis in mediating the pro-proliferative effect of URGCP/URG4 in HCC. Additionally, when we examined the cellular localization of FOXO3a expression within the cell, we noted a decrease in nuclear FOXO3a expression in URGCP/URG4-overexpressing cells (Figure S5A), and a concomitant increase in URGCP/URG4-silenced cells (Figure S5B). This data suggests that the transcriptional activity of FOXO3a might be affected by altered expression of URGCP/URG4.

## Discussion

The data presented in this report suggests a pivotal role of URGCP/URG4, an HBx-upregulated gene, in the progression of HCC. This conclusion is supported by several lines of evidence: first, URGCP/URG4 was found to be overexpressed in all HCC cell lines tested, and in a large proportion of clinical HCC samples. Second, knocking down URGCP/URG4 inhibited proliferation of HCC cells in vitro and in vivo, with the proliferative effect of URGCP/URG4 being is associated with Akt-mediated phosphorylation of FOXO3a that in turn stimulated the cell cycle. Third, the expression level of URGCP/URG4 protein in HCC tissues significantly correlated with the clinical staging of disease and reduced survival time of HCC patients.

In HBV-associated HCC, the viral HBx protein has been recognized as an important factor contributing to enhanced cell proliferation and survival [25], [26]; however, the mechanisms underlying the proliferative and anti-apoptotic effects of HBx remain to be clarified. As a trans-activating factor in HBV-infected liver cells, HBx has been found to contribute to transcriptional regulation of a number of downstream genes, such as URG4, 7, 11, 12 and 19 [18], [27]–[30]. One of these proteins, URG4, referred to here as URGCP/URG4, has previously been found to stimulate HepG2 cell growth and survival, suggesting that URGCP/URG4 might function as a hepatocarcinogenic gene [18]. Such a potential oncogenic function of endogenous URGCP/URG4 in HCC, however, had not previously been studied in vivo, and the molecular pathway via which URGCP/URG4 acts was unknown. Our results show that knockdown of URGCP/URG4 effectively decreases the phosphorylation of FOXO3a and subsequently inhibits G1-S transition, through upregulation of the CDK inhibitors p21^Cip1^ and p27^Kip1^, while downregulating cyclin D1, thus providing new insights into the role of URGCP/URG4 in HCC development and progression associated with HBV or HBx. In parallel with the above findings, we have also found that URGCP/URG4 is strongly expressed in highly proliferative lesions of human HCC, further underscoring the importance of URGCP/URG4 as a modulator of cell cycle regulators during oncogenesis and/or progression of HCC in human patients.

It is of note that FOXO3a, a member of the forkhead box class O (FOXO) transcription factor family, has been found to be functionally important in the development of human HCC [31], [32]. Additionally, to the general understanding that this Akt-regulated transcription factor is overactivated and thereby plays an important role in human cancers [33]–[35], interestingly, the FOXO3a transcription factor has been found to enhance HBV replication by binding viral enhancers and directly activating viral transcription [36]. In the current study, our data suggests a possible regulatory role for URGCP/URG4 in FOXO3a expression, which has been identified as an HBx-induced gene. In this regard, future studies should investigate whether HBV infection and replication in hepatocytes, and altered FOXO3a activity, could form a feedback loop to promote HCC development and progression.

In agreement with the established significance of Akt in HCC, our data also indicates that the FOXO3a phosphorylation induced by URGCP/URG4 is associated with increased Akt activation. It has been shown from in vitro functional studies that the Akt pathway is a pivotal mediator in the control of HCC cellular invasion and motility [37], [38], and the degree of Akt phosphorylation correlates with vascular invasion as well as intrahepatic metastasis of HCC [39], [40]. Furthermore, it has also been suggested that Akt phosphorylation is a risk factor for early disease recurrence and poor prognosis in HCC patients [41]. In the current study, we show that knockdown of URGCP/URG4 could induce a profound reduction in Akt phosphorylation. These data provide evidence for a regulatory effect of URGCP/URG4 on Akt phosphorylation, and also link the URGCP/URG4-triggered Akt signaling cascade with the highly proliferative phenotype of HCC cells. Nevertheless, how URGCP/URG4 stimulates the phosphorylation of Akt remains to be investigated.

Studies have shown that for HCC treatment strategies, such as surgical resection and liver transplantation, there is a longer survival time associated with patients who have a single HCC tumor nodule <5 cm in diameter, or with patients carrying no more than 3 nodules with each smaller than 3 cm in diameter, giving rise to a reported 5-year survival of 50 to 70% [42]. Difficulties in determining the post-surgery clinical outcome of patients with early-stage disease, however, remains challenging. In our stratified analysis, we find that URGCP/URG4 expression is of prognostic value for OS in early-stage HCC patients. In our test cohort, patients with high levels of URGCP/URG4 expression, including those at early stages of HCC (TNM stage I-II, tumor size <3 cm), display a relatively low OS, even as compared with patients with later stages of disease. It is particular of note, that while patients whose AFP level was not accurately predictive for prognosis, URGCP/URG4 levels could indicate differential survival times among these patients, suggesting that URGCP/URG4 might represent a candidate biomarker to distinguish different OS in those patients with normal serum AFP levels. Given these intriguing results, it would be of great interest to evaluate whether serologic URGCP/URG4 can be of early diagnostic value in HCC.

Given the retrospective nature of the current study, and the inherent limitations of this type of analysis, as well as our inclusion of patients with only resectable tumors, a larger population-based prospective study will be required to definitively establish the link between URGCP/URG4 expression and progression or prognosis of HCC. Furthermore, due to the high incidence of HBV in the Chinese population studied, it is unclear whether URGCP/URG4 also plays a role in HBV-negative HCC. Therefore, further studies exploring the significance of URGCP/URG4 in HBV-negative HCC patients are also warranted. Within the context of these limitations, we conclude that our study has identified URGC/URG4 as a novel oncogenic factor and prognostic indicator in HCC, as well as characterizing the possible mechanism underlying URGCP/URG4’s oncogenic potential. The identification of URGCP/URG4 as an important mediator of HCC oncogenesis also raises the possibility of targeting this molecule for therapeutic purposes.

## Supporting Information

Figure S1 **URGCP/URG4 is elevated in HCC.** Representative IHC analyses of URGCP/URG4 expression in adjacent non-cancerous tissues (ANT) and HCC specimens of different clinical stages (A). Statistical quantification of the average MOD values of URGCP/URG4 staining between adjacent non-cancerous tissues and HCC specimens of different clinical stages (B). The data indicates that the MOD of URGCP/URG4 staining increases as HCC progresses to higher clinical stages. Kaplan-Meier analysis of OS in 278 cases based on URGCP/URG4 expression in HCC clinical subgroups. (C) AFP level could not separate patients with different OS in the study cohort. (D) Compared with the high-URGCP/URG4 expression group, the OS was significantly higher in the low-URGCP/URG4 expression group for patients with either normal AFP levels (≤400 ng/ml; left panel) or with elevated AFP levels (>400 ng/ml; right panel; ). Error bars represent SD from three independent experiments. *P<0.05.
(TIF)

Figure S2 **Upregulation of URGCP/URG4 promotes proliferation of HCC cells.** Western blotting analysis of URGCP/URG4 expression in indicated cells (A). Representative micrographs of crystal violet stained cell colonies (B). Effect of URGCP/URG4 overexpression on the growth of HCC cell lines Hep3B and QGY-7703; MTT assays revealed that URGCP/URG4-transfected cells proliferated more rapidly than vector-control cells (C). The upregulation of URGCP/URG4 promoted the anchorage-independent growth ability of HCC cells; representative micrographs (left panel) and quantification of colonies that contained more than 50 cells (middle panel) or were larger than 0.1 mm (right panel) were scored (D). (E) Representative micrographs (left panel;100× magnification) and quantification of BrdU incorporating-cells after transduced with URGCP/URG4 or control vector. GAPDH was used as a loading control for all Western blots. Each bar represents the mean of three independent experiments. *P<0.05.
(TIF)

Figure S3 **URGCP/URG4 induces proliferation through increasing the proportion of S phase cells.** Flow cytometric analysis of indicated HCC cells transduced with URGCP/URG4 or control vector (left panel), and quantification of G0/G1 and S stage cells in indicated HCC cells (right panel; A). Xenografted HCC experiments using NOD/SCID mice; QGY-7703/vector cells and QGY-7703/URGCP/URG4 cells were injected into the groins of mice; Xenografted tumor nodules excised from experimental mice are pictured (B). Tumor volumes were measured on the indicated days; representative graphs of tumor growth and mean tumor weights 5 weeks after inoculation are shown (C). (D) Quantification of the expression of URGCP/URG4 and Ki-67 in HCC lesion (n = 278). All data are shown as mean ± SD. Each bar represents the mean of three independent experiments. *P<0.05.
(TIF)

Figure S4 **URGCP/URG4 is essential for HCC cell proliferation.** Representative micrographs (left panel; 100× magnification) and quantification (right panel) of BrdU incorporating-cells after transduced with URGCP/URG4 RNAis and RNAi vector (A). Flow cytometric analysis of the cell cycle in indicated HCC cells transduced with URGCP/URG4 RNAis or RNAi vector cells in Hep3B (B) and QGY-7703(C) cells.
(TIF)

Figure S5 **Cellular translocation of FOXO3a upon URGCP/URG4-overexpression and silencing.** Cytoplasmic and nuclear levels of FOXO3a in QGY-7703 and Hep3B cells transduced with URGCP/URG4 or control vector was analyzed by WB (A). Cytoplasmic and nuclear levels of FOXO3a in QGY-7703 and Hep3B cells after transduced with URGCP/URG4 RNAis or RNAi vector was analyzed by WB (B).
(TIF)

Table S1 **Clinicopathological characteristics of clinical samples and expression of URGCP/URG4 in liver cancer.**
(DOCX)

Table S2 **Correlation between URGCP/URG4 expression and clinicopathologic characteristics of liver cancer patients.**
(DOCX)

Table S3 **Spearman analysis of correlation between URGCP/URG4 and clinicopathological factors.**
(DOCX)

Table S4 **Univariate and multivariate analyses of various rognostic parameters in patients with liver cancer by Cox-regression analysis.**
(DOCX)

Materials and Methods S1. Plasmids. RNA extraction, reverse transcription (RT) and real-time PCR
3-(4, 5-Dimethyl-2-thiazolyl)-2, 5-diphenyl-2H-tetrazolium bromide (MTT) assay
Anchorage-independent growth ability assay
Bromodeoxyuridine labeling and immunofluorescence
Colony formation assay
Flow cytometry
Preparation of Cytoplasmic and Nuclear Fractions
Western blotting.
(DOC)

## References

[1] Parkin DM, Bray F, Ferlay J, Pisani P (2005). Global cancer statistics, 2002.. CA Cancer J Clin.

[2] Llovet JM, Burroughs A, Bruix J (2003). Hepatocellular carcinoma.. Lancet.

[3] Chen CJ, Yang HI, Su J, Jen CL, You SL (2006). Risk of hepatocellular carcinoma across a biological gradient of serum hepatitis B virus DNA level.. JAMA.

[4] Aravalli RN, Steer CJ, Cressman EN (2008). Molecular mechanisms of hepatocellular carcinoma.. Hepatology.

[5] Sasaki Y, Yamada T, Tanaka H, Ohigashi H, Eguchi H (2006). Risk of recurrence in a long-term follow-up after surgery in 417 patients with hepatitis B- or hepatitis C-related hepatocellular carcinoma.. Ann Surg.

[6] Lai EC, Fan ST, Lo CM, Chu KM, Liu CL (1995). Hepatic resection for hepatocellular carcinoma. An audit of 343 patients.. Ann Surg.

[7] Ercolani G, Grazi GL, Ravaioli M, Del Gaudio M, Gardini A (2003). Liver resection for hepatocellular carcinoma on cirrhosis: univariate and multivariate analysis of risk factors for intrahepatic recurrence.. Ann Surg.

[8] Kinugasa H, Nouso K, Takeuchi Y, Yasunaka T, Onishi H (2011). Risk factors for recurrence after transarterial chemoembolization for early-stage hepatocellular carcinoma.. J Gastroenterol.

[9] Qiu J, Huang P, Liu Q, Hong J, Li B (2011). Identification of MACC1 as a novel prognostic marker in hepatocellular carcinoma.. Journal of Translational Medicine.

[10] El-Serag HB (2004). Hepatocellular carcinoma: recent trends in the United States.Gastroenterology.

[11] Sherman M (2008). Recurrence of hepatocellular carcinoma.. N Engl J Med.

[12] Giuseppe Manghisi, Silvana Elba, Ascanio Mossa, Antonio Giorgio, Vincenza Aloisio (1998). A new prognostic system for hepatocellular carcinoma: a retrospective study of 435 patients: the Cancer of the Liver Italian Program (CLIP) investigators.. Hepatology.

[13] Kudo M, Chung H, Osaki Y (2003). Prognostic staging system for hepatocellular carcinoma (CLIP score): its value and limitations, and a proposal for a new staging system, the Japan Integrated Staging Score (JIS score).. J Gastroenterol.

[14] Hsu CY, Hsia CY, Huang YH, Su CW, Lin HC (2010). Selecting an Optimal Staging System for Hepatocellular Carcinoma – Comparison of 5 Currently Used Prognostic Models.. Cancer.

[15] Kaseb AO, Hassan MM, Lin E, Xiao L, Kumar V (2011). V-CLIP: Integrating Plasma Vascular Endothelial Growth Factor Into a New Scoring System to Stratify Patients With Advanced Hepatocellular Carcinoma for Clinical Trials.. Cancer.

[16] Wang JH, Changchien CS, Hu TH, Lee CM, Kee KM (2008). The efficacy of treatment schedules according to Barcelona Clinic Liver Cancer staging for hepatocellular carcinoma: survival analysis of 3,892 patients.. Eur J Cance.

[17] Koike K, Moriya K, Iino S, Yotsuyanagi H, Endo Y (1994). High-level expression of hepatitis B virus HBx gene and hepatocarcinogenesis in transgenic mice.. Hepatology.

[18] Tufan NL, Lian Z, Liu J, Pan J, Arbuthnot P (2002). Hepatitis Bx antigen stimulates expression of a novel cellular gene, URG4, that promotes hepatocellular growth and survival.. Neoplasia.

[19] Song J, Xie H, Lian Z, Yang G, Du R (2006). Enhanced cell survival of gastric cancer cells by a novel gene URG4.. Neoplasia.

[20] Huang J, Zhu B, Lu L, Lian Z, Wang Y (2009). The expression of novel gene URG4 in osteosarcoma: correlation with patients’ prognosis.. Pathology.

[21] Peng SY, Chou SP, Hsu HC (1998). Association of downregulation of cyclin D1 and of overexpression of cyclin E with p53 mutation, high tumor grade and poor prognosis in hepatocellular carcinoma.. J Hepato.

[22] Ito Y, Matsuura N, Sakon M, Miyoshi E, Noda K (1999). Expression and prognostic roles of the G1-S modulators in hepatocellular carcinoma: p27 independently predicts the recurrence.. Hepatology.

[23] Li J, Zhang N, Song LB, Liao WT, Jiang LL (2008). Astrocyte elevated gene-1 is a novel prognostic marker for breast cancer progression and overall patient survival.. Clin Cancer Res.

[24] Huang H, Tindall DJ (2007). FoxO transcription factors.. J Cell Sci.

[25] Wang WL, London WT, Lega L, Feitelson MA (1991). HBxAg in liver from carrier patients with chronic hepatitis and cirrhosis.. Hepatology.

[26] Benn J, Schneider RJ (1995). Hepatitis B virus HBx protein deregulates cell cycle checkpoint controls.. Proc Natl Acad Sci.

[27] Lian Z, Liu J, Li L, Li X, Clayton M (2006). Enhanced cell survival of Hep3B cells by the hepatitis B x antigen effector, URG11, is associated with upregulation of beta-catenin.. Hepatology.

[28] Du R, Huang C, Bi Q, Zhai Y, Xia L (2010). URG11 mediates hypoxia-induced epithelial-to-mesenchymal transition by modulation of E-cadherin and β-catenin.. Biochem Biophys Res Commun.

[29] Fan R, Li X, Du W, Zou X, Du R (2011). Adenoviral-mediated RNA interference targeting URG11 inhibits growth of human hepatocellular carcinoma.. Int J Cancer.

[30] Pan J, Lian Z, Wallett S, Feitelson MA (2007). The hepatitis B x antigen effector, URG7, blocks tumour necrosis factor alpha-mediated apoptosis by activation of phosphoinositol 3-kinase and beta-catenin.. J Gen Viro.

[31] Lu M, Ma J, Xue W, Cheng C, Wang Y (2009). The expression and prognosis of FOXO3a and Skp2 in human hepatocellular carcinoma.. Pathol Oncol Res.

[32] Kim BC (2008). FoxO3a mediates transforming growth factor-beta1-induced apoptosis in FaO rat hepatoma cells.. BMB Rep.

[33] Brognard J, Clark AS, Ni Y, Dennis PA (2001). Akt/protein kinase B is constitutively active in non-small cell lung cancer cells and promotes cellular survival and resistance to chemotherapy and radiation.. Cancer Re.

[34] Andre F, Nahta R, Conforti R, Boulet T, Aziz M (2008). Expression patterns and predictive value of phosphorylated Akt in early-stage breast cancer.. Ann Onc.

[35] Sonoda Y, Ozawa T, Aldape KD, Deen DF, Berger MS (2001). Akt pathway activation converts anaplastic astrocytoma to glioblastoma multiforme in a human astrocyte model of glioma.. Cancer Re.

[36] Y Nie (2011). Foxo3a regulates hepatitis b virus replication by the activation of HBV enhancer 1 function.. J Hepatol.

[37] Krasilnikov M, Ivanov VN, Dong J, Ronai Z (2003). ERK and PI3K negatively regulate STAT-transcriptional activities in human melanoma cells: implications towards sensitization to apoptosis.. Oncogene.

[38] Saxena NK, Sharma D, Ding X, Lin S, Marra F (2007). Concomitant activation of the JAK/STAT, PI3K/Akt, and ERK signaling is involved in leptin-ediated promotion of invasion and migration of hepatocellular carcinoma cells.. Cancer Res.

[39] Li W, Tan D, Zhang Z, Liang JJ, Brown RE (2008). Activation of Akt-mTOR-p70S6K pathway in angiogenesis in hepatocellular carcinoma.. Oncol Rep.

[40] Chen JS, Wang Q, Fu XH, Huang XH, Chen XL (2009). Involvement of PI3K/PTEN/AKT/mTOR pathway in invasion and metastasis in hepatocellular carcinoma: Association with MMP-9.. Hepatol Res.

[41] Nakanishi K, Sakamoto M, Yamasaki S, Todo S, Hirohashi S (2005). Akt phosphorylation is a risk factor for early disease recurrence and poor prognosis in hepatocellular arcinoma.. Cancer.

[42] Llovet JM, Bruix J (2008). Novel advancements in the management of hepatocellular carcinoma in 2008.. J Hepatol.

