# Supplementary figures and images for: Upregulator of Cell Proliferation Predicts Poor Prognosis in Hepatocellular Carcinoma and Contributes to Hepatocarcinogenesis by Downregulating FOXO3a
Source: PLoS One. 2012 Jul 16;7(7):e40607. doi: 10.1371/journal.pone.0040607 (PMC3398045; doi:10.1371/journal.pone.0040607)

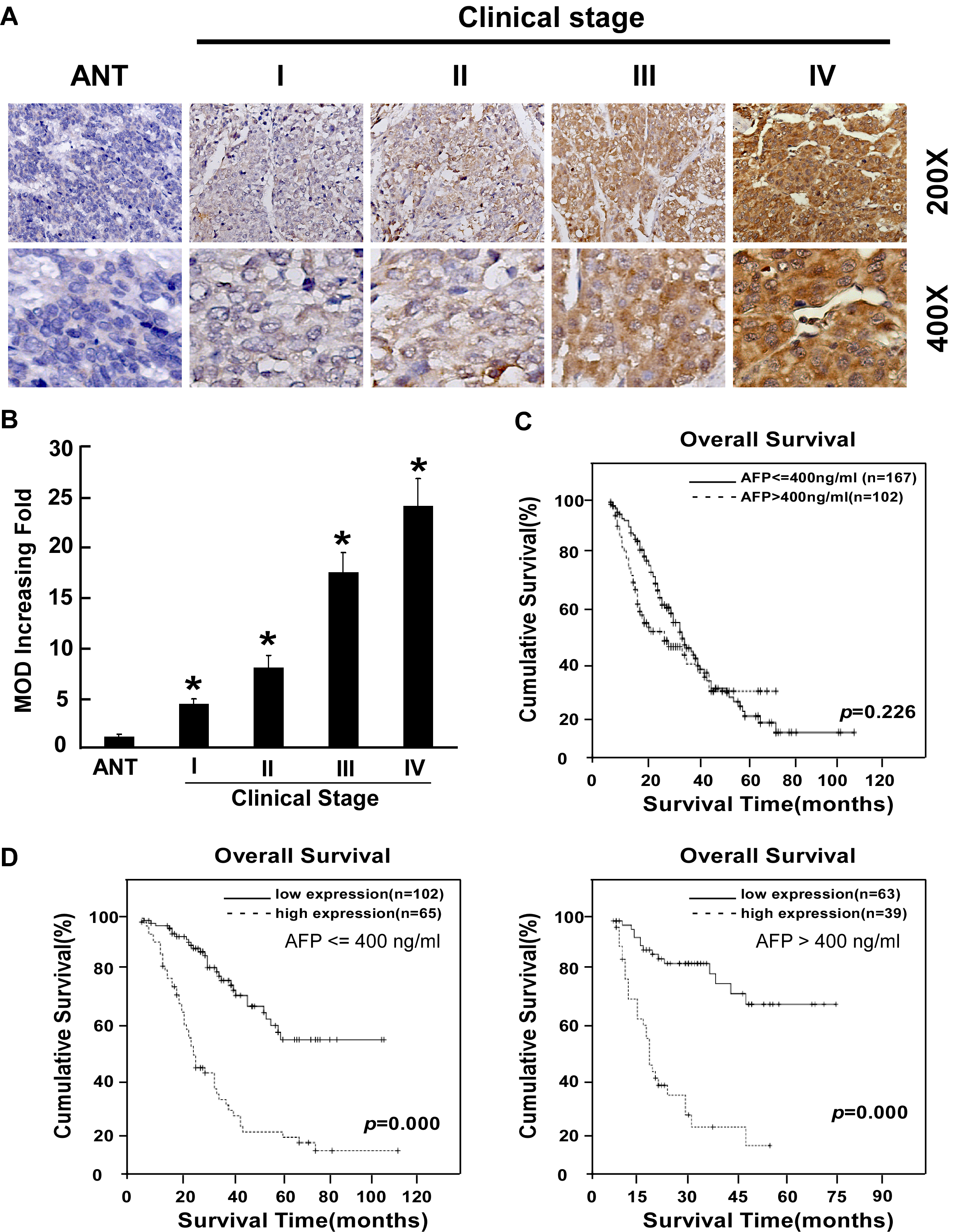

Supplement: Figure S1 — URGCP/URG4 is elevated in HCC. Representative IHC analyses of URGCP/URG4 expression in adjacent non-cancerous tissues (ANT) and HCC specimens of different clinical stages (A). Statistical quantification of the average MOD values of URGCP/URG4 staining between adjacent non-cancerous tissues and HCC specimens of different clinical stages (B). The data indicates that the MOD of URGCP/URG4 staining increases as HCC progresses to higher clinical stages. Kaplan-Meier analysis of OS in 278 cases based on URGCP/URG4 expression in HCC clinical subgroups. (C) AFP level could not separate patients with different OS in the study cohort. (D) Compared with the high-URGCP/URG4 expression group, the OS was significantly higher in the low-URGCP/URG4 expression group for patients with either normal AFP levels (≤400 ng/ml; left panel) or with elevated AFP levels (>400 ng/ml; right panel; ). Error bars represent SD from three independent experiments. *P<0.05. (TIF) [file pone.0040607.s001.tif]

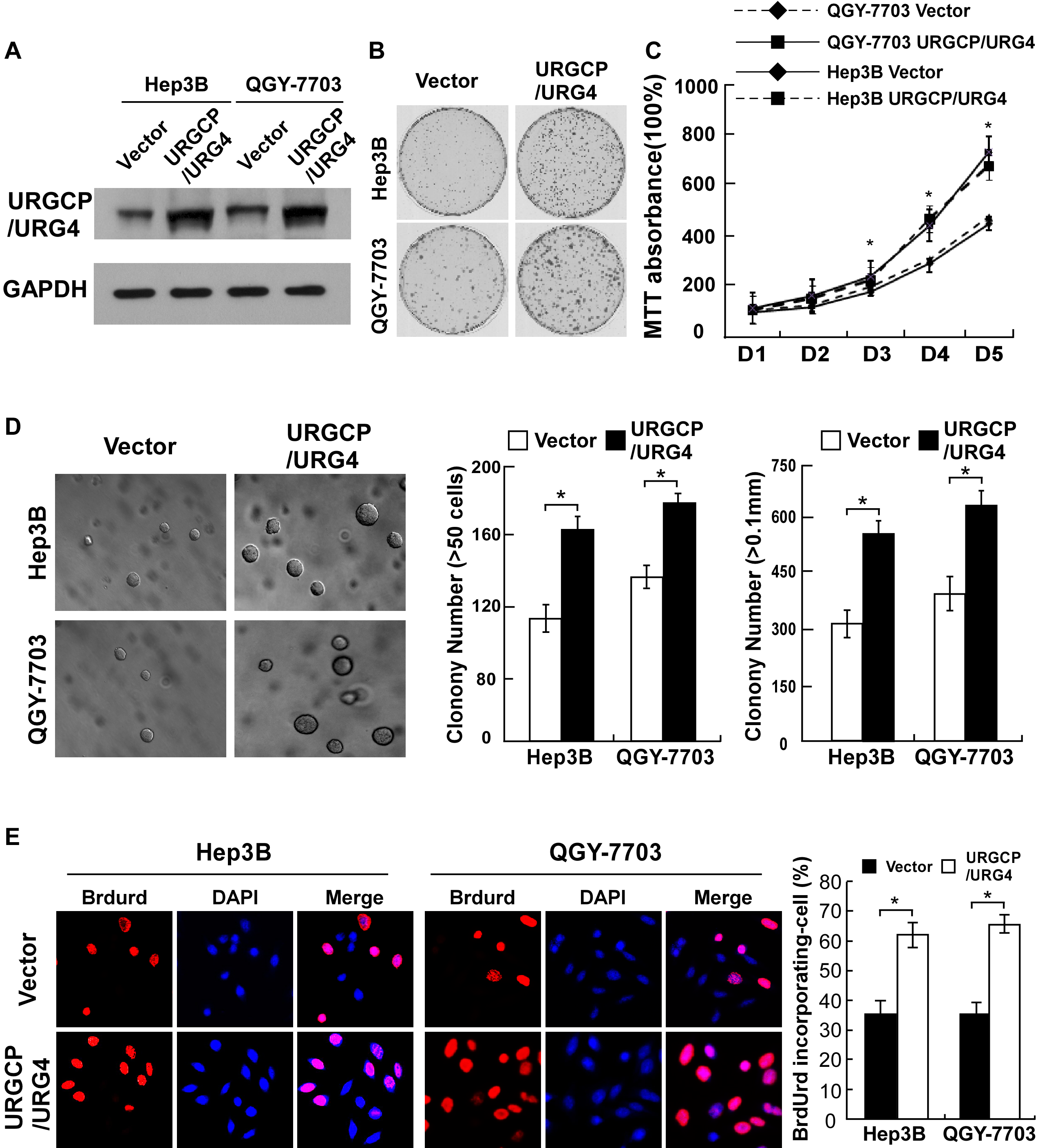

Supplement: Figure S2 — Upregulation of URGCP/URG4 promotes proliferation of HCC cells. Western blotting analysis of URGCP/URG4 expression in indicated cells (A). Representative micrographs of crystal violet stained cell colonies (B). Effect of URGCP/URG4 overexpression on the growth of HCC cell lines Hep3B and QGY-7703; MTT assays revealed that URGCP/URG4-transfected cells proliferated more rapidly than vector-control cells (C). The upregulation of URGCP/URG4 promoted the anchorage-independent growth ability of HCC cells; representative micrographs (left panel) and quantification of colonies that contained more than 50 cells (middle panel) or were larger than 0.1 mm (right panel) were scored (D). (E) Representative micrographs (left panel;100× magnification) and quantification of BrdU incorporating-cells after transduced with URGCP/URG4 or control vector. GAPDH was used as a loading control for all Western blots. Each bar represents the mean of three independent experiments. *P<0.05. (TIF) [file pone.0040607.s002.tif]

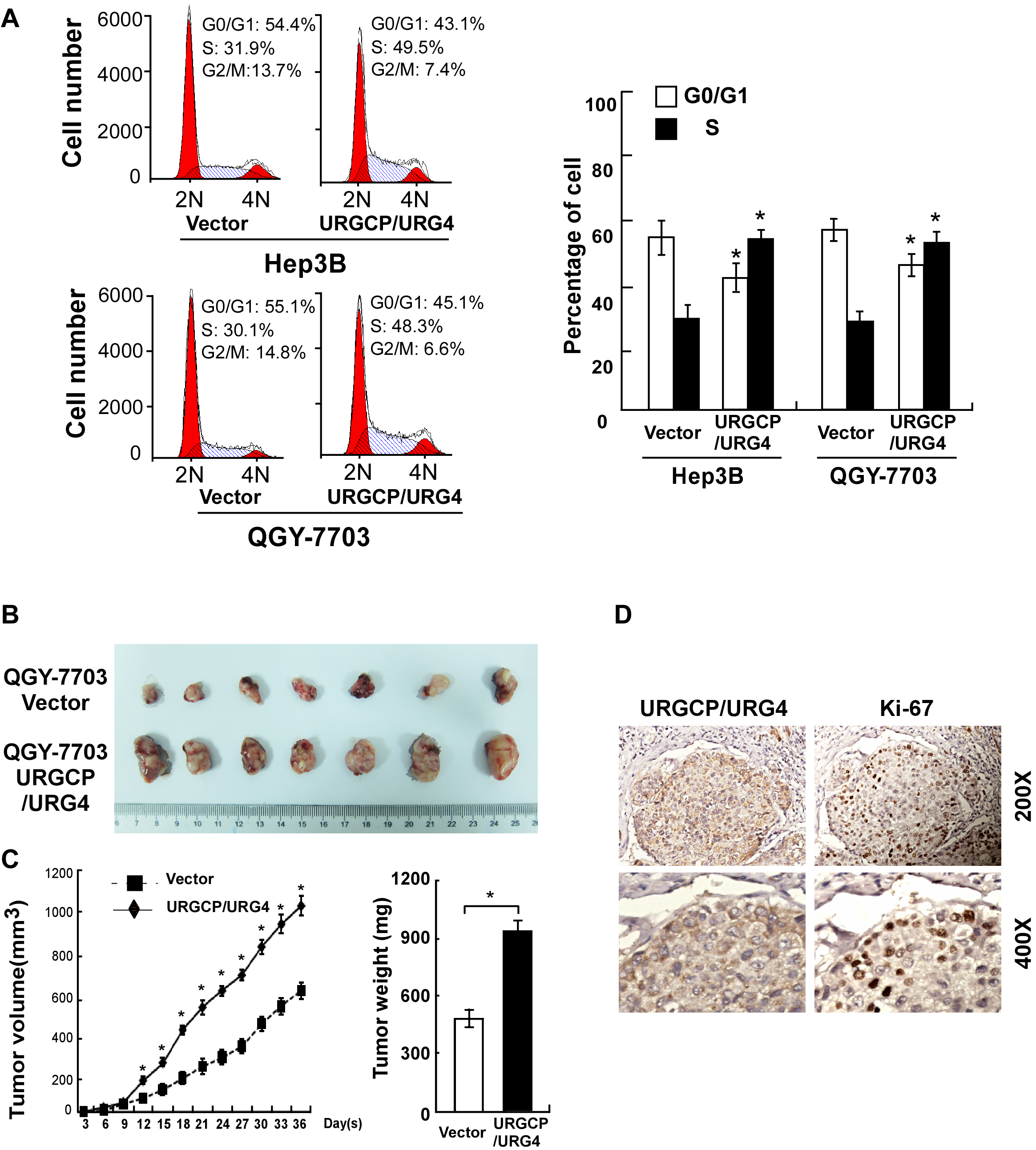

Supplement: Figure S3 — URGCP/URG4 induces proliferation through increasing the proportion of S phase cells. Flow cytometric analysis of indicated HCC cells transduced with URGCP/URG4 or control vector (left panel), and quantification of G0/G1 and S stage cells in indicated HCC cells (right panel; A). Xenografted HCC experiments using NOD/SCID mice; QGY-7703/vector cells and QGY-7703/URGCP/URG4 cells were injected into the groins of mice; Xenografted tumor nodules excised from experimental mice are pictured (B). Tumor volumes were measured on the indicated days; representative graphs of tumor growth and mean tumor weights 5 weeks after inoculation are shown (C). (D) Quantification of the expression of URGCP/URG4 and Ki-67 in HCC lesion (n = 278). All data are shown as mean ± SD. Each bar represents the mean of three independent experiments. *P<0.05. (TIF) [file pone.0040607.s003.tif]

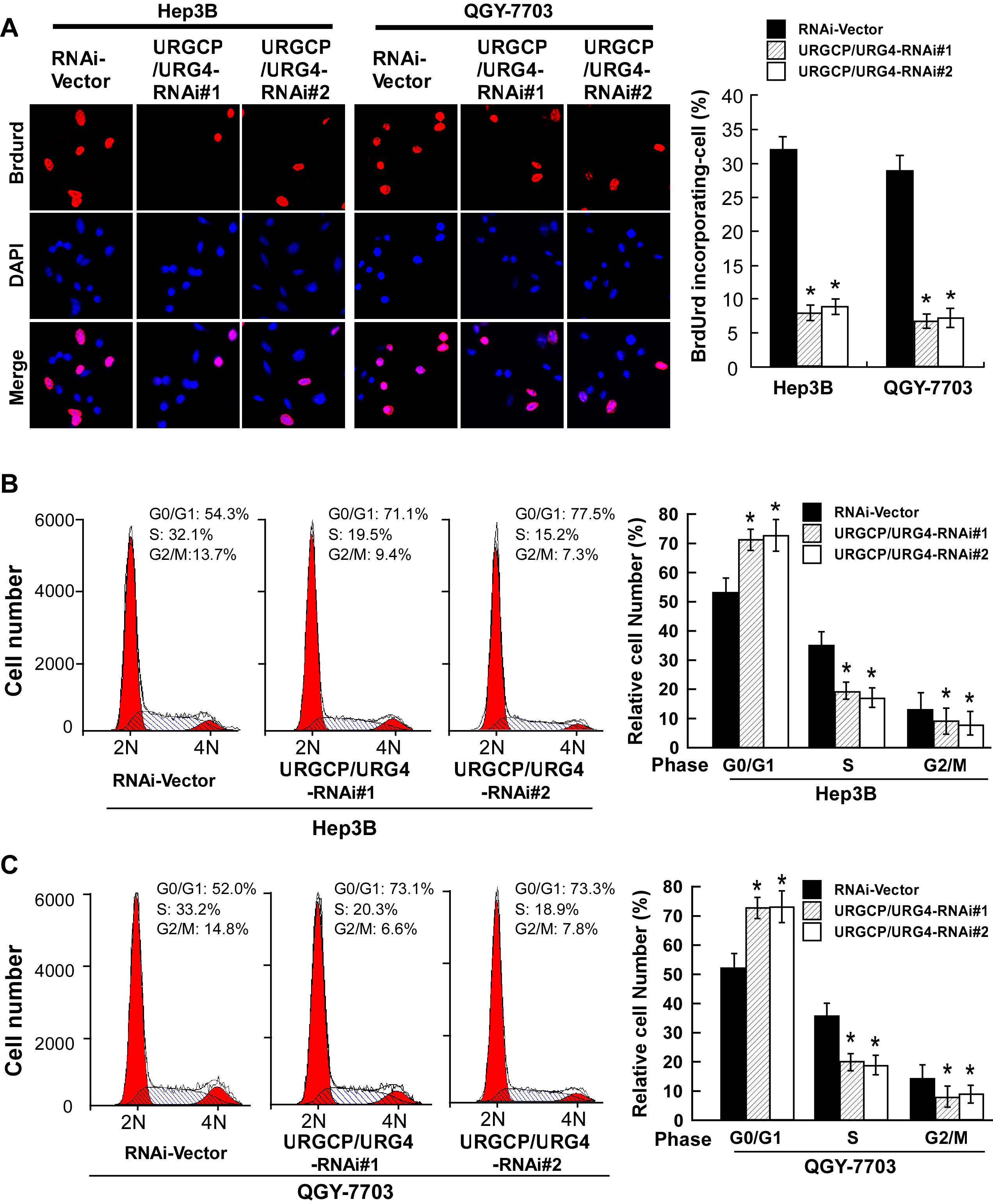

Supplement: Figure S4 — URGCP/URG4 is essential for HCC cell proliferation. Representative micrographs (left panel; 100× magnification) and quantification (right panel) of BrdU incorporating-cells after transduced with URGCP/URG4 RNAis and RNAi vector (A). Flow cytometric analysis of the cell cycle in indicated HCC cells transduced with URGCP/URG4 RNAis or RNAi vector cells in Hep3B (B) and QGY-7703(C) cells. (TIF) [file pone.0040607.s004.tif]

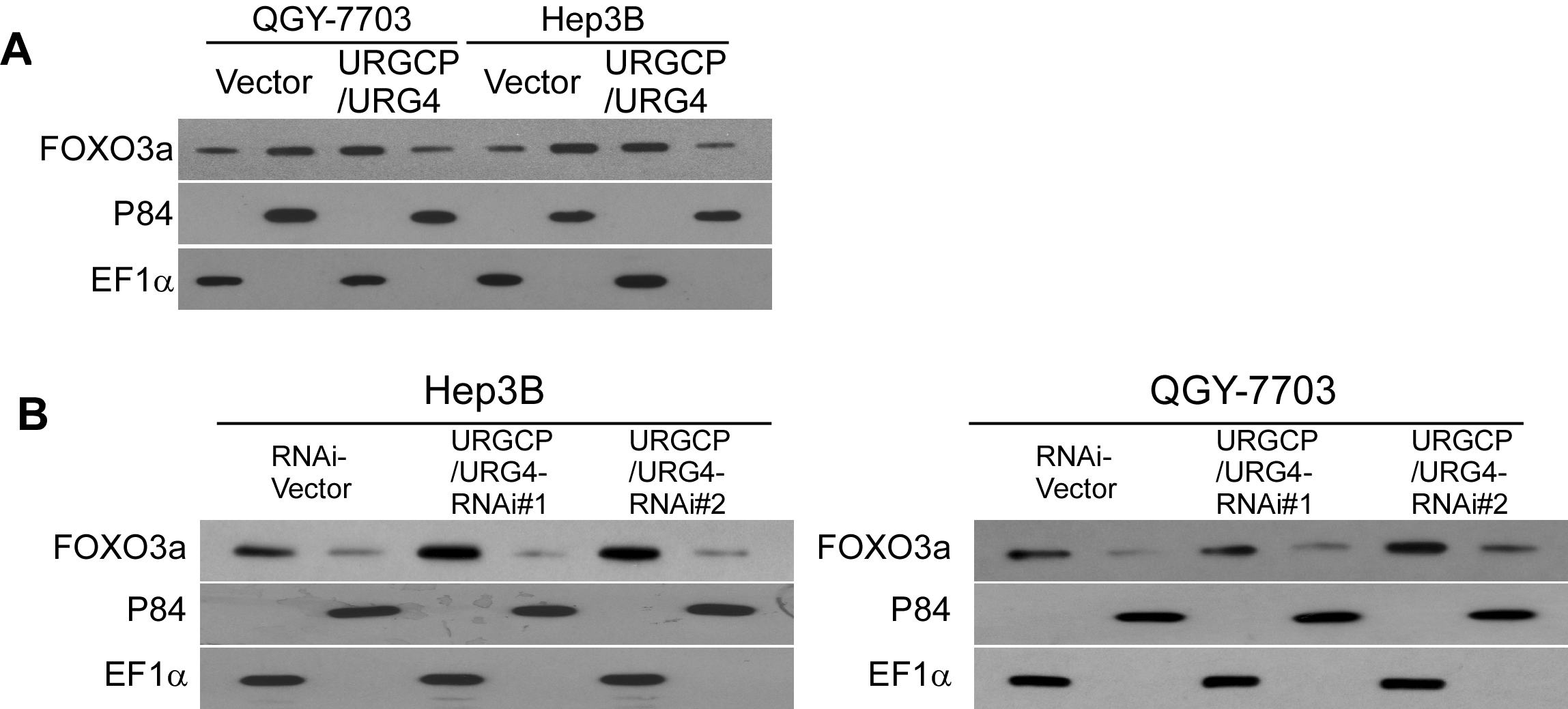

Supplement: Figure S5 — Cellular translocation of FOXO3a upon URGCP/URG4-overexpression and silencing. Cytoplasmic and nuclear levels of FOXO3a in QGY-7703 and Hep3B cells transduced with URGCP/URG4 or control vector was analyzed by WB (A). Cytoplasmic and nuclear levels of FOXO3a in QGY-7703 and Hep3B cells after transduced with URGCP/URG4 RNAis or RNAi vector was analyzed by WB (B). (TIF) [file pone.0040607.s005.tif]
