# Supplementary material for: Upregulator of Cell Proliferation Predicts Poor Prognosis in Hepatocellular Carcinoma and Contributes to Hepatocarcinogenesis by Downregulating FOXO3a
Source: PLoS One. 2012 Jul 16;7(7):e40607. doi: 10.1371/journal.pone.0040607 (PMC3398045; doi:10.1371/journal.pone.0040607)
Supplement: Table S1 — Clinicopathological characteristics of clinical samples and expression of URGCP/URG4 in liver cancer. (DOCX) [file pone.0040607.s006.docx]

**Table S1. Clinicopathological characteristics of clinical samples and expression of URGCP/URG4 in liver cancer**

|  | No. | (%) |
| --- | --- | --- |
| **Age (years)** |  |  |
| <50 | 155 | (55.8) |
| ≥50 | 123 | (44.2) |
| **Gender** |  |  |
| male | 249 | (89.6) |
| female | 29 | (10.4) |
| **TNM classification** |  |  |
| I | 16 | (5.8) |
| II | 195 | (70.1) |
| III | 61 | (21.9) |
| IV | 6 | (2.2) |
| **HBsAg*** |  |  |
| positive | 229 | (87.7) |
| negative | 32 | (12.3) |

| **AFP*** |  |  |
| --- | --- | --- |
| ≥400 **ng/mL** | 102 | (37.9) |
| <400 **ng/mL** | 167 | (62.1) |
| **Tumor size*** |  |  |
| ＞3 cm | 229 | (84.5) |
| ≤3 cm | 42 | (15.5) |
| **Tumor number*** |  |  |
| ＞1 | 94 | (34.2) |
| = 1 | 181 | (65.8) |
| **Vital status (at follow-up)** |  |  |
| Alive | 152 | (54.7) |
| Death due to liver cancer cause | 126 | (45.3) |
| **Expression of URGCP/URG4** |  |  |
| Low expression | 156 | (56.1) |
| High expression | 122 | (43.9) |
|  |  |  |

*Some information of cases were missing, the detail case number were showed in colum.
