# Supplementary material for: Upregulator of Cell Proliferation Predicts Poor Prognosis in Hepatocellular Carcinoma and Contributes to Hepatocarcinogenesis by Downregulating FOXO3a
Source: PLoS One. 2012 Jul 16;7(7):e40607. doi: 10.1371/journal.pone.0040607 (PMC3398045; doi:10.1371/journal.pone.0040607)
Supplement: Table S2 — Correlation between URGCP/URG4 expression and clinicopathologic characteristics of liver cancer patients. (DOCX) [file pone.0040607.s007.docx]

**Table S2. Correlation between URGCP/URG4 expression and clinicopathologic characteristics of liver cancer patients**

|  | **URGCP/URG4** | | **Chi-square**  **test** | |
| --- | --- | --- | --- | --- |
|  |  | |  |  |
| **Characteristic** | **Low** | **High** | **p value** | |
| **Age (＞50 versus**  **≤50 years)** | 77/95 | 46/60 | 0.901 | |
| **Sex (M versus F)** | 149/23 | 100/6 | 0.045 | |
| **TNM stage (I/II**  **versus III/IV)** | 141/31 | 70/36 | 0.003 | |
| **Tumor size (>3cm**  **versus ≤3 cm)** | 138/31 | 91/11 | 0.119 | |
| **AFP (≥400 ng/mL**  **versus ＜400 ng/mL)** | 63/102 | 39/65 | 1.000 | |
| **Tumor number**  **(＞1 versus 1)** | 55/115 | 39/66 | 0.435 | |
| **Vital status (Alive/Death)** | 129/43 | 23/83 | 0.000 | |
| **HBsAg**  **positive/negative** | 143/17 | 86/15 | 0.336 | |
|  | |  |  |  |
