# Supplementary material for: Upregulator of Cell Proliferation Predicts Poor Prognosis in Hepatocellular Carcinoma and Contributes to Hepatocarcinogenesis by Downregulating FOXO3a
Source: PLoS One. 2012 Jul 16;7(7):e40607. doi: 10.1371/journal.pone.0040607 (PMC3398045; doi:10.1371/journal.pone.0040607)
Supplement: Table S3 — Spearman analysis of correlation between URGCP/URG4 and clinicopathological factors. (DOCX) [file pone.0040607.s008.docx]

**Table S3. Spearman analysis of correlation between URGCP/URG4 and clinicopathological factors**

| Variables | URGCP/URG4 expression level | |
| --- | --- | --- |
|  | Spearman Correlation | *p*-Value |
| Survival time | -0.276 | 0.000 |
| Vital status | 0.520 | 0.000 |
| HBsAg | 0.063 | 0.312 |
| Age | 0.038 | 0.527 |
| TNM | 0.160 | 0.007 |
| AFP | 0.000 | 0.995 |
| gender | -0.123 | 0.041 |
| Tumor number | 0.049 | 0.418 |
| Tumor size | 0.059 | 0.333 |

URGCP/URG4 protein expression level in liver cancer significantly correlated with patient survival time (*p* <0.001), the correlation coefficient was -0.276; These results indicate that higher expression levels of URGCP/URG4 correlated with shorter survival time.
