# Supplementary material for: Upregulator of Cell Proliferation Predicts Poor Prognosis in Hepatocellular Carcinoma and Contributes to Hepatocarcinogenesis by Downregulating FOXO3a
Source: PLoS One. 2012 Jul 16;7(7):e40607. doi: 10.1371/journal.pone.0040607 (PMC3398045; doi:10.1371/journal.pone.0040607)
Supplement: Table S4 — Univariate and multivariate analyses of various rognostic parameters in patients with liver cancer by Cox-regression analysis. (DOCX) [file pone.0040607.s009.docx]

**Table S4. Univariate and multivariate analyses of various rognostic parameters in patients with liver cancer by Cox-regression analysis**

|  |  | **Univariate analysis** | | |  | **Multivariate analysis** | | |  |
| --- | --- | --- | --- | --- | --- | --- | --- | --- | --- |
|  | relative risk | | 95% confidence interval | P |  | relative risk | 95%  Confidence  interval | P |  |
|  |  |  |  |  |  |  |  |  |  |
| TNM stage | 1.725 | | 1.234-2.410 | 0.001 |  | 1.506 | 1.083-2.094 | 0.015 |  |
| Tumor size | 2.391 | | 1.251-4.567 | 0.008 |  | 2.016 | 1.052-3.863 | 0.035 |  |
| URGCP/URG4 | 5.132 | | 3.536-7.449 | 0.000 |  | 4.690 | 3.213-6.846 | 0.000 |  |
