# Supplementary material for: Upregulator of Cell Proliferation Predicts Poor Prognosis in Hepatocellular Carcinoma and Contributes to Hepatocarcinogenesis by Downregulating FOXO3a
Source: PLoS One. 2012 Jul 16;7(7):e40607. doi: 10.1371/journal.pone.0040607 (PMC3398045; doi:10.1371/journal.pone.0040607)
Supplement: Materials and Methods S1. Plasmids. — RNA extraction, reverse transcription (RT) and real-time PCR 3-(4, 5-Dimethyl-2-thiazolyl)-2, 5-diphenyl-2H-tetrazolium bromide (MTT) assay Anchorage-independent growth ability assay Bromodeoxyuridine labeling and immunofluorescence Colony formation assay Flow cytometry Preparation of Cytoplasmic and Nuclear Fractions Western blotting. (DOC) [file pone.0040607.s010.doc]

**Supplemental Materials and Methods：**

**Plasmids**

p3x IRS-MLP-luc plasmid was constructed as previously described [1]. The URGCP/URG4 expression construct was generated by subcloning PCR-amplified full-length human URGCP/URG4 cDNA into the pMSCV-retro-puro vector (Promega, Madison, WI) using forward primer 5’-CCAGATCTACCATGGCGTCGC CCGGGCATTC-3’ and reverse primer 5’-GCCGAATTCTCACAGCCGTCTCACCAGC T-3’. For depletion of URGCP/URG4, two human siRNA sequences were cloned into the pSuper-retro-puro plasmid (Promega, Madison, WI) to generate pSuper-retro-URGCP/URG4-RNAi(s), respectively, and the sequences were RNAi#1: ACCAAAGACTTGCCCTGGAATT and RNAi#2: GCGCAACAC AAACCTGAGATT (synthesized by Invitrogen). Retroviral production and infection were performed as described previously [1]. Sequences of FOXO3a siRNAs were: siRNAi#1-AATGTGACATGGAGTCCATTA, and siRNAi#2-GAGCTCTTGGTGGATCATC TT (synthesized by Invitrogen). Transfection of FOXO3a siRNAs was performed using the Lipofectamine 2000 reagent (Invitrogen) according to the manufacturer’s instruction. The reporter plasmid for quantitatively detecting the transcriptional activity of FOXO was generated in the pGL3-Enhancer plasmid (Promega, Madison, WI, USA) as described previously [1].

**RNA extraction, reverse transcription (RT) and real-time PCR**

Total RNA from cultured cells was extracted using the Trizol reagent (Invitrogen, Carlsbad, CA) as the manufacturer instructed. cDNAs were amplified and quantified in ABI Prism 7500 Sequence Detection System (Applied Biosystems, Foster City, CA) using dye SYBR Green I (Molecular Probes, Invitrogen, CA Carlsbad, CA). The primers designed using the Primer Express v 2.0 software (Applied Biosystems) and the primer sequences are provided as following: URGCP/URG4, forward, 5’- CTTCATCCTGAGTCCCTACCG-3’ and reverse, 5’-GCCGT TCTGCTGCATTCG-3’; p21^Cip1^, forward, 5’-CGATGCCAACCTCCTCAACGA-3’, and reverse, 5’-TCGCAGACCTCCAGCATCCA-3’; and p27 ^Kip1^, forward, 5’-TGCAACCGACG ATTCTTCTACTCAA-3’, and reverse, 5’-CAAGCAGTGATGTATCTGATAAACAAGGA-3’; Cyclin D1, forward, 5’- AACTACCTGGACCGCTTCCT-3’, and reverse, 5’-CCACTTGAGC TTGTTCACCA-3’. Expression data were normalized to the geometric mean of housekeeping gene GAPDH (forward, 5’-ACCACAGTCCATGCCATCAC-3’ and reverse, 5’-TCCACCA C CCTG TTGCTGTA -3’) to control the variability in expression levels and calculated as 2^-[(^*^C^t* ^of URGCP/URG4 or p21 or p27) – (^*^C^t* ^of^ *^GAPDH^*)], where C_t_ represents the threshold cycle for each transcript.

**3-(4, 5-Dimethyl-2-thiazolyl)-2, 5-diphenyl-2H-tetrazolium bromide (MTT) assay**

Cells were seeded in 96-well plates at a density of (1×10^3^/well). At each time point, cells were stained with 100 μl sterile MTT dye (0.5 mg/ml, Sigma) for 4 h at 37 ^o^C, followed by removal of the culture medium and addition of 150 μl of dimethyl sulphoxide (Sigma, St. Louis, MO, USA). The absorbance was measured at 570 nm, with 655 nm as the reference wavelength. All experiments were performed in triplicates.

**Anchorage-independent growth ability assay**

One thousand cells were trypsinized and suspended in 2 ml complete medium plus 0.3% agar (Sigma, Saint Louis, MI). The agar-cell mixture was plated on top of a bottom layer of 1% agar completed medium mixture. At 10 days, viable colonies that contained more than 50 cells or were larger than 0.1 mm were counted. The experiment was performed for three independently times for each cell line.

**Bromodeoxyuridine labeling and immunofluorescence**

Cells were plated on coverslips (Fisher, Pittsburgh, PA). After 24 hours, cells were incubated with bromodeoxyuridine (BrdUrd) for 1 h and stained with anti-BrdUrd antibody (Upstate, Temecula, CA) according to the manufacturer’s instruction. Gray level images were acquired under a laser scanning microscope (Axioskop 2 plus, Carl Zeiss Co. Ltd., Jena, Germany).

**Colony formation assay**

Cells were plated at density of 1×10^3^ cells per dish and cultured for 7 days. The colonies were stained with 1% crystal violet for 10 minutes after fixation with 10% formaldehyde for 5 min.

**Flow cytometry**

Cells were harvested, washed with cold PBS and processed for cell cycle analysis using flow cytometry. Briefly, the cells were fixed in 75% ethanol and stored at −20°C for later analysis. The fixed cells were centrifuged at 800 rpm and washed with cold PBS twice. RNase A (20 μg/ml final concentration) and propidium iodide staining solution (50 μg/ml final concentration) was added to the cells and incubated for 30 min at 37°C in the dark. One hundred thousand cells were analyzed using a FACSC alibur instrument (BD Biosciences, San Jose, CA) equipped with CellQuest 3.3 software. ModFit LT 3.1 trial cell cycle analysis software was used to determine the percentage of cells in the different phases of the cell cycle.

**Preparation of Cytoplasmic and Nuclear Fractions**

Cells were scraped with cold PBS and resuspended in HLB buffer (10 mM HEPES [pH 7.9], 10 mM KCl, 1.5 mM MgCl2, 0.5 mM DTT). The cells were then Dounce-homogenized and centrifuged at 1000 × g for 5 min at 4ºC. The supernatant (cytoplasmic fraction) was stored at–80°C until use. The nuclei in the pellet were isolated by centrifugation and resuspended in nuclear extraction buffer (20 mM HEPES [pH 7.9], 420 mM NaCl, 1.2 mM MgCl_2_, 0.2 mM EDTA, 25% glycerol) for 30 min at 4°C. After centrifugation at 15,000 × g for 30 min at 4ºC, the supernatant (nuclear fraction) was stored at –80°C.

**Western blotting**

Western blotting was performed according to standard methods, using anti-URGCP/URG4 (abcam, UK), anti-Akt (Cell Signaling, Danvers, MA), anti-phosphorylated-Akt (Cell Signaling, Danvers, MA), anti-GSK-3β (Cell Signaling, Danvers, MA), anti-phosphorylated-GSK-3β (Cell Signaling, Danvers, MA), anti-phosphorylated-Rb (Cell Signaling, Danvers, MA), anti-Rb (Cell Signaling, Danvers, MA), anti-total FOXO3a and anti-phosphorylated-FOXO3a (Ser^253^) antibodies (Cell Signaling, Danvers, MA), and anti-p27 ^Kip1^ and anti-p21^Cip1^ antibodies (BD, Franklin Lakes, New Jersey). Blotted membranes were stripped and re-probed with an anti-GAPDH antibody (Sigma, Saint Louis, MI) as a loading control.

**Supplemental Figure Legends**

Figure S1. URGCP/URG4 is elevated in HCC. Representative IHC analyses of URGCP/URG4 expression in adjacent non-cancerous tissues (ANT) and HCC specimens of different clinical stages (A). Statistical quantiﬁcation of the average MOD values of URGCP/URG4 staining between adjacent non-cancerous tissues and HCC specimens of different clinical stages (B). The data indicates that the MOD of URGCP/URG4 staining increases as HCC progresses to higher clinical stages. Kaplan-Meier analysis of OS in 278 cases based on URGCP/URG4 expression in HCC clinical subgroups. (C) AFP level could not separate patients with different OS in the study cohort. (D) Compared with the high-URGCP/URG4 expression group, the OS was significantly higher in the low-URGCP/URG4 expression group for patients with either normal AFP levels (≤400 ng/ml; left panel) or with elevated AFP levels (>400 ng/ml; right panel; ). Error bars represent SD from three independent experiments. * *P* < 0.05.

Figure S2. Upregulation of URGCP/URG4 promotes proliferation of HCC cells. Western blotting analysis of URGCP/URG4 expression in indicated cells (A). Representative micrographs of crystal violet stained cell colonies (B). Effect of URGCP/URG4 overexpression on the growth of HCC cell lines Hep3B and QGY-7703; MTT assays revealed that URGCP/URG4-transfected cells proliferated more rapidly than vector-control cells (C). The upregulation of URGCP/URG4 promoted the anchorage-independent growth ability of HCC cells; representative micrographs (left panel) and quantification of colonies that contained more than 50 cells (middle panel) or were larger than 0.1 mm (right panel) were scored (D). (E) Representative micrographs (left panel;100× magnification) and quantification of BrdU incorporating-cells after transduced with URGCP/URG4 or control vector. GAPDH was used as a loading control for all Western blots. Each bar represents the mean of three independent experiments. * *P* < 0.05.

Figure S3. URGCP/URG4 induces proliferation through increasing the proportion of S phase cells. Flow cytometric analysis of indicated HCC cells transduced with URGCP/URG4 or control vector (left panel), and quantification of G0/G1 and S stage cells in indicated HCC cells (right panel; A). Xenografted HCC experiments using NOD/SCID mice; QGY-7703/vector cells and QGY-7703/URGCP/URG4 cells were injected into the groins of mice; Xenografted tumor nodules excised from experimental mice are pictured (B). Tumor volumes were measured on the indicated days; representative graphs of tumor growth and mean tumor weights 5 weeks after inoculation are shown (C). (D) Quantification of the expression of URGCP/URG4 and Ki-67 in HCC lesion (n = 278). All data are shown as mean ± SD. Each bar represents the mean of three independent experiments. * *P* < 0.05.

Figure S4. URGCP/URG4 is essential for HCC cell proliferation. Representative micrographs (left panel; 100× magnification) and quantification (right panel) of BrdU incorporating-cells after transduced with URGCP/URG4 RNAis and RNAi vector (A). Flow cytometric analysis of the cell cycle in indicated HCC cells transduced with URGCP/URG4 RNAis or RNAi vector cells in Hep3B (B) and QGY-7703(C) cells.

Figure S5. Cellular translocation of FOXO3a upon. URGCP/URG4-overexpression and silencing. Cytoplasmic and nuclear levels of FOXO3a in QGY-7703 and Hep3B cells transduced with URGCP/URG4 or control vector was analyzed by WB (A). Cytoplasmic and nuclear levels of FOXO3a in QGY-7703 and Hep3B cells after transduced with URGCP/URG4 RNAis or RNAi vector was analyzed by WB (B).

**Supplemental reference:**

1. Tang ED, Nunez G, Barr FG, et al. Negative regulation of the forkhead transcription factor FKHR by Akt. J Biol Chem 1999; 274:16741-16746.
